# Clostridial Neurotoxins: Mechanism of SNARE Cleavage and Outlook on Potential Substrate Specificity Reengineering

**DOI:** 10.3390/toxins2040665

**Published:** 2010-04-13

**Authors:** Thomas Binz, Stefan Sikorra, Stefan Mahrhold

**Affiliations:** Institut für Biochemie, OE 4310, Medizinische Hochschule Hannover, 30623 Hannover, Germany; Email: sikorra.stefan@mh-hannover.de (S.S.); mahrhold.stefan@mh-hannover.de (S.M.)

**Keywords:** botulinum neurotoxin, tetanus toxin, SNARE, zinc protease, enzyme engineering

## Abstract

The clostridial neurotoxin family consists of tetanus neurotoxin and seven distinct botulinum neurotoxins which cause the diseases tetanus and botulism. The extreme potency of these toxins primarily relies not only on their ability to specifically enter motoneurons but also on the activity their catalytic domains display inside presynaptic motoneuronal terminals. Subsequent to neurotoxin binding and endocytosis the catalytic domains become translocated across endosomal membranes and proteolyze unique peptide bonds of one of three soluble *N*-ethylmaleimide-sensitive fusion protein attachment receptors (SNAREs), vesicle associated membrane protein/synaptobrevin, synaptosome associated protein of 25 kDa, or syntaxin. As these substrate proteins are core components of the vesicular membrane fusion apparatus, cleavage of any of the substrate molecules results in the blockade of neurotransmitter release. This review summarizes the present knowledge about the molecular basis of the specific substrate recognition and cleavage mechanism and assesses the feasibility of reengineering catalytic domains to hydrolyze non-substrate members of the three SNARE families in order to expand the therapeutic application of botulinum neurotoxins.

## 1. Introduction

**Figure 1:**
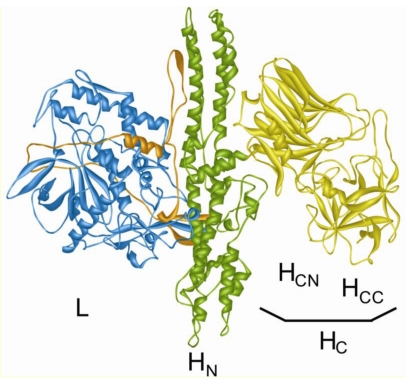
Structure of CNTs. Ribbon representation of BoNT/A. The catalytic (L), translocation (H_N_), and binding domains (H_C_; consisting of H_CC_ and H_CN_) are specified. The H_N_ domain derived loop wrapping around the LC (L) is colored orange.

Clostridial neurotoxins (CNT), a family of closely related bacterial protein toxins, consist of seven antigenically distinguishable botulinum neurotoxins (BoNTs) and tetanus neurotoxin (TeNT). Each CNT is synthesized as a ~150 kDa single chain protein, but subsequently cleaved by specific clostridial or host proteases. Cleavage results in an *N*-terminal ~50 kDa light chain (LC) and a *C*-terminal ~100 kDa heavy chain (HC). Both chains remain attached *via* a single disulfide bond formed between cysteines at the *C*-terminus of the LC and the *N*-terminus of the HC, a peptide loop formed by the *N*-terminal ~55 amino acids of HC that wraps around the LC and further non-covalent interactions (Figure 1). The HC comprises two subunits, a largely α‑helical domain of ~50 kDa at the *N*-terminus, designated H_N_, and a ~50 kDa fragment at the *C*-terminus, H_C_, which is composed of two ~25 kDa domains, a lectin like jelly role domain H_CN_ and a β-trefoil domain H_CC_ (Figure 1).

Poisoning by BoNTs mainly occurs upon oral ingestion and toxin uptake at intestinal epithelial cells, whereas TeNT is produced and released into the circulation by bacteria that germinate in infected tissue lesions. Eventually CNTs reach cholinergic motor nerve terminals, where a four step process is initiated that ultimately renders a synapse inoperative. In order that they can be taken up *via* the recycling pathway of synaptic vesicles (SV), they first bind to complex gangliosides at the surface of nonmyelinated nerve terminals [1]. Requisite for the second step, the endocytosis, is the binding of the toxin to an additional receptor. In case of BoNTs this may generally be an intravesicular portion of a synaptic vesicle transmembrane protein, which becomes transiently exposed on the nerve cell surface during the course of exo- and endocytosis. Recent work had demonstrated that synaptotagmin (Syt)-I and Syt-II act as protein receptors for BoNT/B and G [2,3,4,5] and the synaptic vesicle protein (SV) 2 as receptor for BoNT/A and E [6,7,8], and possibly also BoNT/F [9,10]. Protein receptors have not yet been determined for BoNT/C and D as well as TeNT. The interaction with a ganglioside and the respective protein receptor appears to be mediated by H_CC_ as demonstrated for TeNT and several BoNTs. In contrast, the role of the H_CN_‑domain is still unresolved, although low affinity binding to lipidic membrane components was recently reported for BoNT/A H_CN_ [11]. Intracellular transport subsequent to endocytosis proceeds differently for BoNTs and TeNT. TeNT obviously harnesses a particular protein receptor that directs this toxin to the transcytosis pathway instead of the SV recycling pathway carrying it into the spinal cord, where it has to repeat the first two steps of the four step mechanism of action. Whether in a cholinergic nerve terminal or in a neuron of the spinal cord, next the toxins become exposed to an acidic environment during transport of the endocytosed vesicle which initiates the third step. Acidification triggers a structural rearrangement in H_N_ and the formation of a cation selective channel. This is probably coupled to partial unfolding of the LC, its entry into and transit through the H_N_ channel followed by its refolding in the cytosol [12,13]. Subsequent to reduction of the disulfide bridge the LC is released from the HC. Once in the cytosol the LCs exert their catalytic activity, the molecular mechanisms of which will be discussed in the following sections, and proteolyze core components of the vesicular membrane fusion apparatus. Substrate cleavage leads to a blockade of neurotransmitter release, but to different clinical symptoms for BoNTs *versus* TeNT. BoNTs act inside the motoneuron and thus cause flaccid paralysis, which can result in respiratory failure and death. TeNT poisons inhibitory interneurons to cause just opposite symptoms, spastic paralysis [14].

CNTs are regarded as the most hazardous natural substances known. The 50% lethal doses for susceptible mammals including man amount to approximately one nanogram per kg of body weight [15]. Due to the lack of immunization protection in the population, BoNTs also represent a major bioweapon [16,17]. On the other hand, BoNT/A and B have become widely used therapeutics for the treatment of a variety of neurological disorders with still increasing numbers of indications during the past two decades [18].

## 2. Cleavage of SNAREs

**Figure 2:**
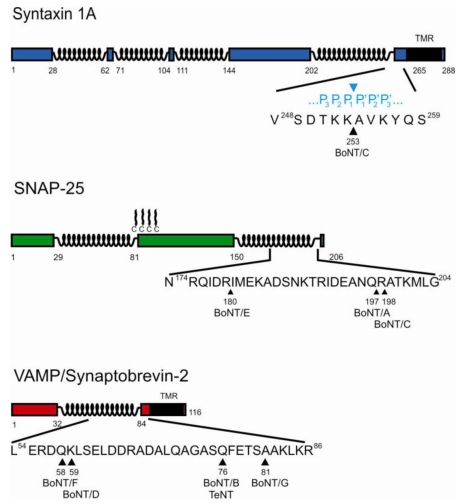
Schematic drawing of the substrates of CNTs and specification of the individual cleavage sites. Syntaxin 1A (top) is a type II membrane protein that contains four α-helical regions. The *C*-terminal α-helical region is involved in SNARE complex formation. Synaptosome associated protein of 25 kDa (SNAP-25, middle) is anchored in the plasma membrane *via* palmitoyl groups that are attached to cysteine residues in the middle of the molecule. Both its α-helical segments become constituent parts of the SNARE complex in the course of membrane fusion. Vesicle associated membrane protein (VAMP)/Synaptobrevin-2 (bottom), a type II membrane protein of synaptic vesicles, completes the coiled-coil four helix SNARE bundle complex *via* inclusion of its central α-helical region to the other three helices. The individual peptide bonds hydrolyzed by CNTs are indicated by arrow heads below the primary structure sections and the *N*-terminal amino acid positions are specified. The substrate positions (P3 to P3’) around the scissile peptide bond as defined by Schechter and Berger [89] are shown above the primary structure section of Syntaxin 1A.

The intracellular mode of action of the neurotoxins was discovered in the early 1990s, initiated by the identification of the Zn^2+^ binding motif, His-Glu-X-X-His, in the primary sequence of TeNT [19] and its conservation in the amino acid sequence of BoNT/A [20,21]. Evidence for a zinc dependent proteolytic mode of action was provided *via* the demonstration of zinc binding for BoNTs [22] and the abolishment of TeNT evoked blockade of neurotransmitter release by classical zinc endoprotease inhibitors [23]. Shortly after, selective searches for substrates among synaptic vesicle proteins led to the identification of the integral synaptic vesicle membrane protein VAMP (vesicle associatedmembrane protein), also termed synaptobrevin, as target for BoNT/B and TeNT [24,25,26]. BoNT/D, F, and G were subsequently also shown to cleave VAMP/synaptobrevin [27,28,29]. However, except for TeNT and BoNT/B, the toxins hydrolyze different peptide bonds. BoNT/D and F cut the neighboring bonds Gln58-Lys59 and Lys59-Leu60, respectively, almost in the middle of the 116-amino acid sized VAMP/synaptobrevin-2, whereas the scissile bond for BoNT/B and TeNT is located 17 amino acids further downstream and that for BoNT/G shifted by additional five residues towards the *C*-terminal transmembrane domain (Figure 2; [25,27,28,29]). The proteolytic activity of the remaining CNTs was found to be directed against proteins of the presynaptic membrane. BoNT/A, C, and E proteolyze SNAP-25 (synaptosomal associated protein of 25 kDa) [27,30,31,32,33]. Again different peptide bonds are attacked. BoNT/A and C hydrolyze neighboring bonds, 9 or 8 amino acids, respectively, away from the *C*-terminus of this 206 residues comprising protein, while BoNT/E cleavage releases the 26 *C*-terminal residues [34,35,36]. The substrate specificity of BoNT/C turned out to be unique, since yet another protein of the presynaptic membrane, syntaxin, had already been identified as substrate [37,38,39]. The hydrolysable peptide bond, Lys253-Ala254, is again located in the *C*-terminal region, 12 amino acids upstream of the *C*-terminal transmembrane domain (Figure 2; [40]).

The CNT substrate proteins are members of larger protein families that are collectively termed SNAREs (soluble *N*-ethylmaleimide-sensitive factor attachment protein receptors). A common feature of all SNAREs is the presence of one (or as an exception two) α-helical domain(s) of ~70 amino acids, the SNARE motif, endowed with the capacity to form coiled-coils. Sets of SNAREs generally build the core for the individual intracellular membrane fusion processes and zippering of such sets of SNAREs *via* their SNARE motifs into parallel four-helix coiled-coil bundles is regarded to provide the driving force for membrane fusion [41,42]. E.g., the mammalian syntaxin 1A, SNAP-25 (comprising two SNARE domains), and VAMP/synaptobrevin-2 are the nerve terminal SNAREs and mediate neurotransmitter release into the synaptic cleft. Consequently, their cleavage results in the blockade of neurotransmission.

Studies about the susceptibility of other mammalian SNAREs toward CNTs revealed that the majority is not hydrolysable. In addition to the established substrates, merely VAMP/synaptobrevin-1, cellubrevin/VAMP-3, syntaxin 2, syntaxin 3, and the murine SNAP‑23 proved to be substrates for the respective CNT serotypes (reviewed in [43]). Susceptibility at least at high LC concentrations can be conferred to some non-substrate SNAREs by replacements of one or two crucial amino acids [36,44,45].

## 3. Catalytic Mechanism

The mode of Zn^2+^-coordination classifies the LCs of CNTs into the gluzincin superfamily among the clan MA of metalloproteases [46,47]. As a characteristic feature of this superfamily, Zn^2+^ is coordinated by two histidine residues of the His-Glu-X-X-His motif, a water molecule, which is bonded to the glutamate residue of the motif, and another glutamic acid residue located about 35 residues downstream in the primary structure. Searches for proteins exhibiting a related tertiary structure revealed that the central portion is structurally similar to a portion of the zinc metalloprotease thermolysin [48]. These similarities are limited to the helix containing the His-Glu-X-X-His motif and a four-stranded β-sheet buttressing the helix. The loop regions connecting the conserved secondary structure elements of the catalytic core are drastically expanded compared to those of thermolysin. In addition, the CNT catalytic core is not involved in substrate binding *via* side-chain side-chain interactions. Substrate specificity is thus imparted by distinct amino- and carboxyl-terminal structural elements flanking the core, which appear to be structurally unique to the CNTs [49], as they contain substrate-binding sites (see below; [50]).

As thermolysin represents the well characterized prototypical member of the gluzincin metalloprotease superfamily, its mode of peptide bond cleavage is presumably to a large extent similar to that of CNTs. In case of thermolysin, catalysis follows a general base-type mechanism [51]. It was proposed that the water molecule is polarized *via* its interaction with Zn^2+^ and the glutamic acid carboxylate group of the His-Glu-X-X-His-motif and can thereby nucleophilically attack the carbonyl carbon of the scissile peptide bond to form an oxyanion. Simultaneously, a proton abstracted from the attacking water is shuttled *via* the carboxyl group of the glutamate to the scissile peptide bond nitrogen, and the glutamate may then stabilize the tetrahedral intermediate by forming a salt bridge with the positively charged amide nitrogen. The negative charge that develops on the carbonyl oxygen atom in the tetrahedral transition state is stabilized by hydrogen bonding interactions with a protonated histidine (position 231) and the hydroxyl group of a tyrosine (position 157). It is assumed that His-231 is retained in proper position and protonated state through a hydrogen bonding interaction with an aspartate (position 226). The protonated amide nitrogen then facilitates C-N-bond disruption and may subsequently receive a second proton derived from the water, possibly again mediated by the glutamic acid carboxylate group [51,52].

**Figure 3:**
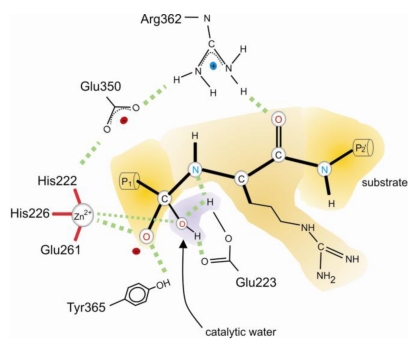
Proposed hydrolytic mechanism of BoNT/A. In the first step, a water molecule polarized by Glu223 and Zn^2+^, nucleophilically attacks the carbonyl carbon of the scissile peptide bond to form an oxyanion. The side chain of Tyr365 is responsible for the stabilization of the negative charge that develops in the tetrahedral transition state on the carbonyl carbon atom. For Arg362 the scenario that proposes interactions between its guanidino group and the carbonyl oxygen of the P1’ amino acid is depicted. Glu350 likely keeps Arg362 and His222 in proper position through ionic interactions. Peptide bond cleavage is likely achieved by two proton transfers from the attacking water mediated by the carboxyl group of Glu223 and results in a protonated amine. The nomenclature used for substrate amino acids is according to Schechter and Berger [89].

The available data on CNTs support a similar mechanism (Figure 3). In fact, the nucleophilic attack of the scissile bond carbonyl carbon by the coordinated polarized water molecule as well as the subsequent relocation of a proton from the attacking water to the scissile peptide bond nitrogen may be ascribed to the action of glutamate of the His-Glu-X-X-His motif as its mutation leads to complete deactivation of the catalytic activity of e.g., BoNT/A [53] and BoNT/E [54]. It has also been discussed that a conserved tyrosine serves as proton donor for the amide nitrogen. If this was true, one would expect a much more severe effect on the hydrolytic activity by its replacement by phenylalanine (see below).

Primary structure alignments of all eight CNTs revealed three conserved residues within the otherwise less conserved region around the zinc coordinating residues of the LCs, *i.e.*, a glutamate, an arginine, and a tyrosine (Figure 3). 

They reside ~120 amino acids downstream from the His-Glu-X-X-His motif and are separated by 11 and 2 amino acids, respectively. Two recently solved crystal structures of LC/A and LC/E bound to non-cleavable short SNAP-25 peptides comprising the respective scissile bonds showed that this tyrosine (position 365 and 350 in LCA and LCE, respectively) lines up in close proximity to the scissile peptide bond [55,56] and might therefore be the functionally equivalent residue to Tyr-157 of thermolysin. Its central role in substrate cleavage was demonstrated for TeNT (position 374) and BoNT/A and E. In BoNT/A and TeNT the substitution with phenylalanine drastically decreased the hydrolytic activity [57,58]. Kinetic analyses for BoNT/A-Y365F revealed a substantial reduction of k_cat_, while Zn^2+^-binding and substrate binding were not significantly affected [57,59]. This finding was interpreted as participation of its side group in the stabilization of the transition state oxyanion [57] and was later on also evidenced by structural studies [56,60]. Substitution of tyrosine with alanine even abolished the enzymatic activity of BoNT/E and TeNT [58,61]. The stronger effect caused by the mutation to alanine might be due to additional important interactions such as interaction of the benzene ring with the P2’ (for explanation see Figure 2) carbonyl group in case of BoNT/E [55].

The counterpart of the thermolysin His-231 might be the above mentioned strictly conserved arginine (position 362 and 347 in LCA and LCE, respectively). Its replacement in BoNT/A by various residues and by alanine in BoNT/E generally led to an approx. 100-fold decrease in k_cat_, while K_M_ remained unaffected [57,59,61]. The difference in free energy for transition state binding of mutated *vs.* wild-type LCA as well as the guanidino group positioning in the vicinity of the scissile peptide bond led to the suggestion that this residue might also be involved in oxyanion binding in the transition state [57,60]. Alternatively, recently determined co-crystal structures of LC/A and LC/E bound to short non-cleavable SNAP-25 derived peptides suggested an H-bond interaction between the Arg362 guanidino group and the carbonyl oxygen of the P1’ position [55,56].

CNTs presumably also possess an analog to Asp-226 of thermolysin. A glutamate, the third of the above mentioned strictly conserved residues in the vicinity of the zinc coordinating residues, occupies a similar position in all CNTs [62]. Removal of its negative charge dramatically diminished the hydrolytic activity of BoNT/A and E [57,61]. Beside a corresponding role in securing the proper positioning and the protonated state of the guanidino group of the conserved arginine it adds to the negative electrostatic potential and, at variance with thermolysin, it appears to be important for orienting one of the Zn^2+^ coordinating histidines at the active site [57,61].

## 4. Mode of Substrate Recognition

**Figure 4:**
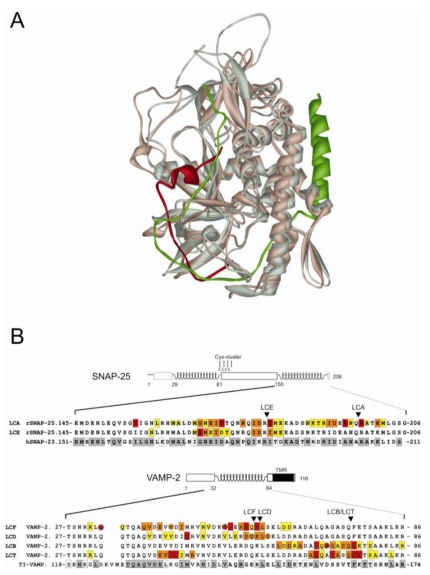
Substrate recognition of CNTs. **(a)** Cartoon illustration of the mode of substrate binding of LCA and LCF. Superposition of ribbon diagrams of LCA (gray) bound to SNAP-25 (residues 152-202; green) and LCF (tan) bound to VAMP/synaptobrevin-2 (residues 32-58; red). **(b)** Individual substrate requirements of LCs.Amino acid sequences of VAMP-2 and TI-VAMP (lower panel) as well as those of rat SNAP-25 and human SNAP-23 (upper panel) were aligned. The scissile bonds of LCs are indicated by arrow heads. Conserved residues are depicted by gray boxes in the sequence of SNAP-23 and TI-VAMP, whereas similar residues are given by open boxes. Residues shown in red, orange, or yellow boxes in separate lines for the various LCs indicate that removal of their side group decreases cleavage by the respective LC (given left) by more than 75%, 50%, or 25%, respectively. Residues highlighted by circles using the same color code depict to what extent introduction or reversal of charges affects cleavage. Data from the following publications were incorporated into this scheme: [44,65,70,73,81,90,91,92,93]. In case of divergent data on mutants, averaged values are shown.

CNTs display unique substrate selectivity in contrast to the broad specificity of conventional proteases. Initial studies employing various sized peptides comprising the individual cleavage sites revealed that extended substrate sections of about 30 amino acids are required for optimal catalytic activity of VAMP/synaptobrevin-2 cleaving [29,63,64,65,66,67] and SNAP-25 cleaving [36,68] CNTs. Furthermore, the substrate-length requirements varied with the CNT serotype. For example, BoNT/B and TeNT, although hydrolyzing the identical peptide bond, were shown to be dependent upon different sized (40 and 62 residues, respectively) peptides [64]. Complementary cleavage studies on substrate point mutants indicated that the enzymatic activity of all CNTs is dependent upon substrate interactions remote from the cleavage site and thus not upon structural constraints [36,45,62,67,69,70,71,72]. Since that time the involvement of each VAMP/synaptobrevin-2 and SNAP-25 residue in LC binding has been analyzed by systematic approaches for all LC serotypes but BoNT/G and C [44,73]. The collected results verified the above mentioned findings and led to an in-depth map of crucial substrate residues and provided evidence for the resistance of SNARE isoforms like human SNAP-23 or TI-VAMP/VAMP-7 *versus* CNTs (Figure 4B).

The recognition model involving substrate/enzyme interactions remote from the active site proved to be compatible with crystal structure data obtained for a catalytically inactive BoNT/A LC bound to the carboxyl terminal half (residues 141-204) of SNAP-25 [50]. The co-crystal revealed an extensive interface between protease and substrate. The SNAP-25 segment Gln152 to Met202 was found to wrap around most of the LC’s periphery (Figure 4A), and 19 amino acids thereof were proposed to establish side-chain/side-chain contacts with the protease distributed along the whole interface. It is feasible that SNAP-25 binding initiates at the most distal 21-residue segment (known as the α-exosite), which forms a distorted α-helix, and is followed by the formation of successive additional interactions *via* anchor points that together direct the path of the substrate into the LC’s active site [50]. A comparison of LC structures in their free and substrate bound states provided evidence that regions near the active site cavity underwent structural rearrangements on substrate interaction. In particular, the loop around amino acid position 250 switches from an open to a closed conformation in order to facilitate substrate binding at the catalytic centre [50]. These data argue for an induced fit recognition mechanism for BoNT/A. The extraordinary substrate specificity of CNTs may thus be explained by the requirement for remote interaction sites and their proper spatial relationship to the specific cleavage site.

The co-crystal structure of LCA bound to SNAP-25 does not allow for the definition of the LC pockets involved in the binding of substrate residues near the scissile bond, as a double mutant of the active site was used to render the enzyme inactive [50]. A number of recently solved co-crystal structures and docking studies for LCA with small-molecule inhibitors [74,75] and SNAP-25 derived peptides [56,60,76,77,78] contributed to fill this information gap. In particular, crystals of LCA bound to non-cleavable six-residue SNAP-25 peptides, one containing Gln197-Arg-Ala-Thr-Lys-Met202, whereas the authentic P1 Gln-197 was replaced with arginine in the second peptide, allowed for the unequivocal definition of the pockets S1-S5’ [56]. The importance of several of these suggested LC pocket forming amino acids was corroborated in parallel studies by enzymatic characterization of LC mutants [59,79]. Similar information about interactions at the active site has also been collected for LCE. Here, a co-crystal structure of the non-cleavable four-residue SNAP-25 peptide, Arg180-Ile-Met-Glu183, that represents P1-P3’, could be solved and the LC residues of the S1-S3’ pockets be delineated [55]. The LCE residues forming the S1’ and S2’ pockets had been suggested earlier based on the enzymatic characterization of LCE mutants [80]. Moreover, in the latter study, LC residues representing constituents of the S2 and S3 pockets have also been suggested.

Structure data about the mode of VAMP/synaptobrevin-2 recognition by LCF have recently also been achieved [81]. In this case, the co-crystallization of two, 37- and 32-residue, VAMP mimicking peptide inhibitors ending in D-cysteine instead of Gln-58 at the P1 position succeeded. The direction of substrate binding was found to be similar to that of thermolysin and to those observed for SNAP-25 peptides and LCA and LCE. The paths how the inhibitors bind along the LCF surface are basically similar to that of SNAP-25 on LCA, though loops protrude differently. In addition, VAMP/synaptobrevin-2 exhibits each one short segment of β-sheet and α-helix conformation, whereas SNAP-25 is extended in the entire matchable area (residues 168–197). However, the SNAP-25/LCA interface is longer; a 21-residue distorted α-helix (residues 147–167) joins in *N*-terminal direction and forms major interactions with LCA (Figure 4A; [50]). Like the SNAP-25/LCA interaction, side-chain/side-chain amino acids contacts between VAMP/synaptobrevin-2 and LCF are suggested to exist along the whole interface. Cleavage studies using mutants confirmed the importance of some of the LC and substrate residues and underscored that contacts remote from the scissile bond are critical for an efficient substrate recognition process [44,81]. Thus, the course of VAMP/synaptobrevin-2 binding by LCF might occur similarly to that outlined for SNAP-25 and LCA. Due to the nature of the LCF inhibitors used, the co-crystal structure, however, does not provide information about interactions taking place downstream from the P1 site. Results of cleavage studies that employed mutated VAMP/synaptobrevin-2 also coincide with a similar recognition mechanism, as the P1’-P2’ residues proved to be important for substrate cleavage, whereas residues located further downstream probably do not contribute or are far less critical for substrate recognition (Figure 4B; [44,65,67,70]).

Superposition of the substrate-free LCF structure onto the LCF-VAMP/synaptobrevin-2 structure led to the detection of major side chain rotamer variation around the active site. These facilitate proper substrate binding by opening the active site and allow for salt bridges with the P3 and P2 residues [81]. The steric changes in LCF upon substrate binding are also compatible with an induced fit recognition mechanism as proposed for BoNT/A.

## 5. Reengineered CNT Catalytic Domains to Target Non-substrate SNAREs

Twenty years ago BoNTs became an approved therapeutic for the treatment of the neurological disorders strabismus, blepharospasm, and hemifacial spasm. Since then their application range steadily increased and includes today further medical conditions like axillary hyperhydrosis or even cosmetic use. However, the clinical application of BoNTs is restricted to disorders that rely on hyperactivity of cholinergic neurons, due to the toxins’ tropism for these neurons and the preferential cleavage of neuronal SNAREs.

These limitations can now probably be bypassed, owing to the elucidation of the molecular structure of BoNTs and the increasing information about structure-function relationships. Consequently, attempts were undertaken to retarget BoNT activity to specific neurons or non-neuronal cells by replacing their cell binding subunit, H_C_. For example, the *Erythrina cristagalli* lectin, selectively recognizing galactose containing carbohydrates as present on nociceptive afferents, was chemically conjugated to purified L-H_N_ of BoNT/A. This conjugate was shown to affect nociceptive neurons, which opens up the possibility to develop retargeted BoNTs for the treatment of chronic pain [82,83]. Furthermore, a fully recombinant fusion protein consisting of L-H_N_ of BoNT/C and the epidermal growth factor was designed. This construct was reported to inhibit mucin secretion from the human respiratory epithelial cell line A549 and might thus have the potential to treat mucus hypersecretion in asthma and chronic obstructive pulmonary disease [84]. The latter example demonstrates that the intracellular action of BoNTs can in principle be harnessed to interfere with therapeutically relevant release processes of non-neuronal cells. However, if the release process is driven by SNAREs that are not susceptible, the LC has to be reengineered in order to be able to attack the desired SNARE.

Such a re-targeting has recently been achieved for LCE. Its enzymatic activity could be extended to human SNAP-23 by a single amino acid replacement [85]. Within the identified interacting segment of the natural substrate human SNAP-25, Met167-Asp186 [73], human SNAP-23 differs in six amino acids (Figure 4B). The exchange of Asp179 by Lys185 in the P2 position of SNAP-25 had been recognized to be most critical for cleavage by BoNT/E [36,73], probably because it forms a salt bridge with Lys224 of the respective LCE S2 pocket [80]. Consequently, the resistance of human SNAP-23 might predominantly be due to repulsion of the P2 residue Lys185. Chen and Barbieri therefore replaced the LCE S2 pocket residue Lys224 with aspartic acid. *In vitro* cleavage assays showed that the mutated LCE exhibited a hydrolysis rate in the range of SNAP-25 cleavage by wild-type LCE *versus* the otherwise non-cleavable human SNAP-23; K_M_/k_cat_ was nine-fold reduced [85]. Furthermore, LCE-Lys224Asp decreased IL-8 and mucin secretion in TGF-α stimulated HeLA cells [85].

**Figure 5:**
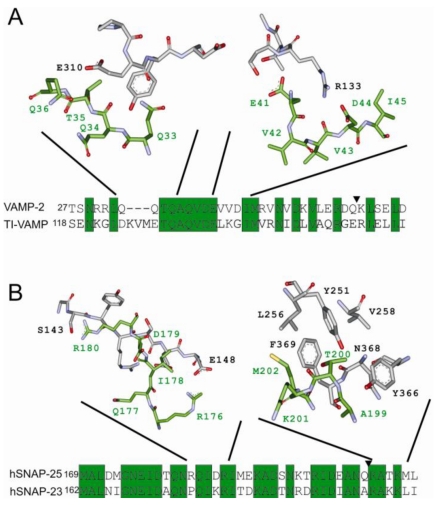
Mode of substrate recognition in selected LC binding pockets. Stick representations of LC (gray) and substrate (green) amino acid residues in their steric configuration at selected binding pockets. Amino acid variations between established substrates and non‑cleavable isoforms are depicted by amino acid alignments. Scissile bonds for LCF and LCA are indicated by arrowheads. **(a)** VAMP/synaptobrevin-2 binding by LCF at two selected binding sites, whose configuration might contribute to inefficient TI-VAMP interaction. (**b)** SNAP-25 binding by LCA at two elected binding sites, whose configuration might contribute to inefficient hSNAP-23 interaction.

BoNT/A is currently the most attractive CNT serotype in terms of medical application, due to its pronounced longevity *versus* other BoNTs and the existing practice of its handling. It appears feasible that LCA could also be re-targeted to human SNAP-23. Promising starting points are an enlargement of or/and introduction of a negatively charged side group into the S3’ pocket to accommodate the SNAP-23 P3’ Lys206, or to adapt the hydrophobic S5’ pocket for the exchange of SNAP-25 Met202 by Leu208 in SNAP-23 (Figure 5; [50,56]). In addition, as compensation for the lost important salt bridge between Arg176 and LCA Glu148, likely constituting a major anchor point for the progress of SNAP-25 binding, new salt bridges might be generated at other positions, e.g., by replacing Val304 or Ser143 with aspartic acid to form salt bridges with hSNAP-23 Lys185 and Arg186, respectively.

The non-cleavable TI-VAMP ([86]; also called VAMP-7) might also be an interesting target. It is known to be involved in processes mediating neurite outgrowth, synaptic transmission, plasma membrane remodelling, and lysosomal secretion (reviewed in [87]). Based on already available data (see above), adaptation in LCF could be conducted to endow it with specificity for this SNARE. The removal of the negative charge of Glu315 or/and Glu310 might avoid charge dependent repulsion by TI-VAMP Asp124 and Glu128, respectively. Likewise, exchange of Arg133 might eliminate interference with TI-VAMP Lys137 (Figure 5). Enlargement of the LCF S1’ pocket, by means of two glutamic acid to aspartic acid substitutions could facilitate P1’ interaction with Arg153, the corresponding residue of VAMP-2 Lys59. However, “high throughput” screening systems are certainly required, in particular if LC pockets must be restructured fundamentally involving simultaneously mutations of several amino acids. Possibly, yeast is suitable for screening of LC mutants, as exemplified recently for a viral protease [88].

If LC re-targeting succeeded one may wish that the re-targeted LC loses its activity *versus* the original substrate, in case that the original and the new substrate are co-expressed in the desired target cell. Therefore, in addition to mutations which enable cleavage of the new substrate, LC amino acids must be identified and replaced whose mutations exclusively impair cleavage of the original substrate.
